# Correction: The structural role of SARS-CoV-2 genetic background in the emergence and success of spike mutations: The case of the spike A222V mutation

**DOI:** 10.1371/journal.ppat.1010995

**Published:** 2022-11-23

**Authors:** Tiziana Ginex, Clara Marco-Marín, Miłosz Wieczór, Carlos P. Mata, James Krieger, Paula Ruiz-Rodriguez, Maria Luisa López-Redondo, Clara Francés-Gómez, Roberto Melero, Carlos Óscar Sánchez-Sorzano, Marta Martínez, Nadine Gougeard, Alicia Forcada-Nadal, Sara Zamora-Caballero, Roberto Gozalbo-Rovira, Carla Sanz-Frasquet, Rocío Arranz, Jeronimo Bravo, Vicente Rubio, Alberto Marina, The IBV-Covid19-Pipeline, Ron Geller, Iñaki Comas, Carmen Gil, Mireia Coscolla, Modesto Orozco, José Luis Llácer, Jose-Maria Carazo

In the Financial Disclosure, the number for the grant awarded by EPIDNA is incorrect. The correct EPIDNA grant number is 894489.
