# Supplementary material for: The structural role of SARS-CoV-2 genetic background in the emergence and success of spike mutations: The case of the spike A222V mutation
Source: PLoS Pathog. 2022 Jul 11;18(7):e1010631. doi: 10.1371/journal.ppat.1010631 (PMC9302720; doi:10.1371/journal.ppat.1010631)
Supplement: S1 Text — Figs A-N and Tables A-G. The structural role of SARS-CoV-2 genetic background in the emergence and success of spike mutations: the case of the spike A222V mutation (DOCX) [file ppat.1010631.s001.docx]

**SUPPLEMENTARY INFORMATION**

**The structural role of SARS-CoV-2 genetic background in the emergence and success of spike mutations: the case of the spike A222V mutation**

Tiziana Ginex,^1§^ Clara Marco-Marín,^2,7§^ Miłosz Wieczór,^3§^ Carlos P. Mata,^4,8§^ James Krieger,^4§^ Paula Ruiz-Rodriguez,^5§^ Maria Luisa López-Redondo,^2^ Clara Francés-Gómez,^5^ Roberto Melero,^4^ Carlos Óscar Sánchez-Sorzano,^4^ Marta Martínez,^4^ Nadine Gougeard,^2,7^ Alicia Forcada-Nadal,^2,7^ Sara Zamora-Caballero,^2^ Roberto Gozalbo-Rovira,^2^ Carla Sanz-Frasquet,^2^ Rocío Arranz,^4^ Jeronimo Bravo,^2^ Vicente Rubio,^2,7^ Alberto Marina,^2,7^ The IBV-Covid19-Pipeline,^2^ Ron Geller,^5^ Iñaki Comas,^2,6^ Carmen Gil,^1^ Mireia Coscolla,^5^ Modesto Orozco,^3,9^ José Luis Llácer,^2,7^ and José-Maria Carazo^4^*

**The IBV-Covid19-Pipeline:** Laura Villamayor, Carolina Espinosa, Anmol Adhav, Maria del Pilar Hernández-Sierra, Rafael Ruiz-Partida, Jesus Rodríguez-Díaz.

**List of the IBV-Covid19-Pipeline members that did not directly contribute to the manuscript**: Susana Masiá, Francisca Gallego, Monica Escamilla-Aguilar, Antonio Rubio-Del-Campo, Lidia Orea-Ordóñez, Alonso Felipe, Borja Saez-De la Fuente, Guilherme Dim, Alba Iglesias-Ceacero, Francisco Del Caño-Ochoa, Javier Mancheño, Santiago Ramón-Maiques.

**Table of Content:**

**Figs**

*Page S3* **Fig A.** Temporal distribution of Delta sub-lineages.

*Page S4* **Fig B.** Recurrent emergence of S:A222V in different SARS-CoV-2 scenarios in the G clade

*Page S5* **Fig C.** Protein purification and quality analysis.

*Page S5* **Fig D.** Thermofluor assays (means of three replicas) of the indicated SARS-CoV-2 S protein mutants.

*Page S6-7* **Fig E.** Kinetic analysis of spike interaction to ACE2 measured by biolayer interferometry (BLI).

*Page S7* **Fig F.** Cryo-EM data of [S:A222V + S:D614G].

*Page S8-9* **Fig G.** Cryo-EM image processing workflow for S:D614G.

*Page S10-11* **Fig H.** Cryo-EM image processing workflow for [S:A222V + S:D614G].

*Page S12* **Fig I.** Cryo-EM of S:D614G.

*Page S13* **Fig J.** Comparison of the two structures at the region where S:A222V mutation is located.

*Page S14* **Fig K.** Continuous population densities along the PC1.

*Page S15* **Fig L.** PCA of whole experimental S:D614G spike structures excluding 2-up conformations.

*Page S16* **Fig M.** PCA of whole S:D614G PDB structures including 2-up conformations.

*Page S17* **Fig N.** RMSD analysis.

**Tables**

*Page S18* **Table A.** Frequency of sequences with A222V for the different PANGO lineages.

*Page S19* **Table B.** Cryo-EM data collection, refinement and validation statistics.

*Page S20* **Table C.** Interaction between RBDs and NTDs domains from different subunits.

*Page S21* **Table D.** Detailed description of the 3-down (DDD) and 1-up (UDD) models of the SARS-CoV-2 spike mutants simulated in this study.

*Page S22* **Table E.** Mutational free energy analysis.

*Page S23* **Table F.** Experimental structures of the SARS-CoV-2 S:D614G spike used in PCA.

*Page S24* **Table G.** Oligonucleotides used in this study.

*Page S25* **References**

**Figs**


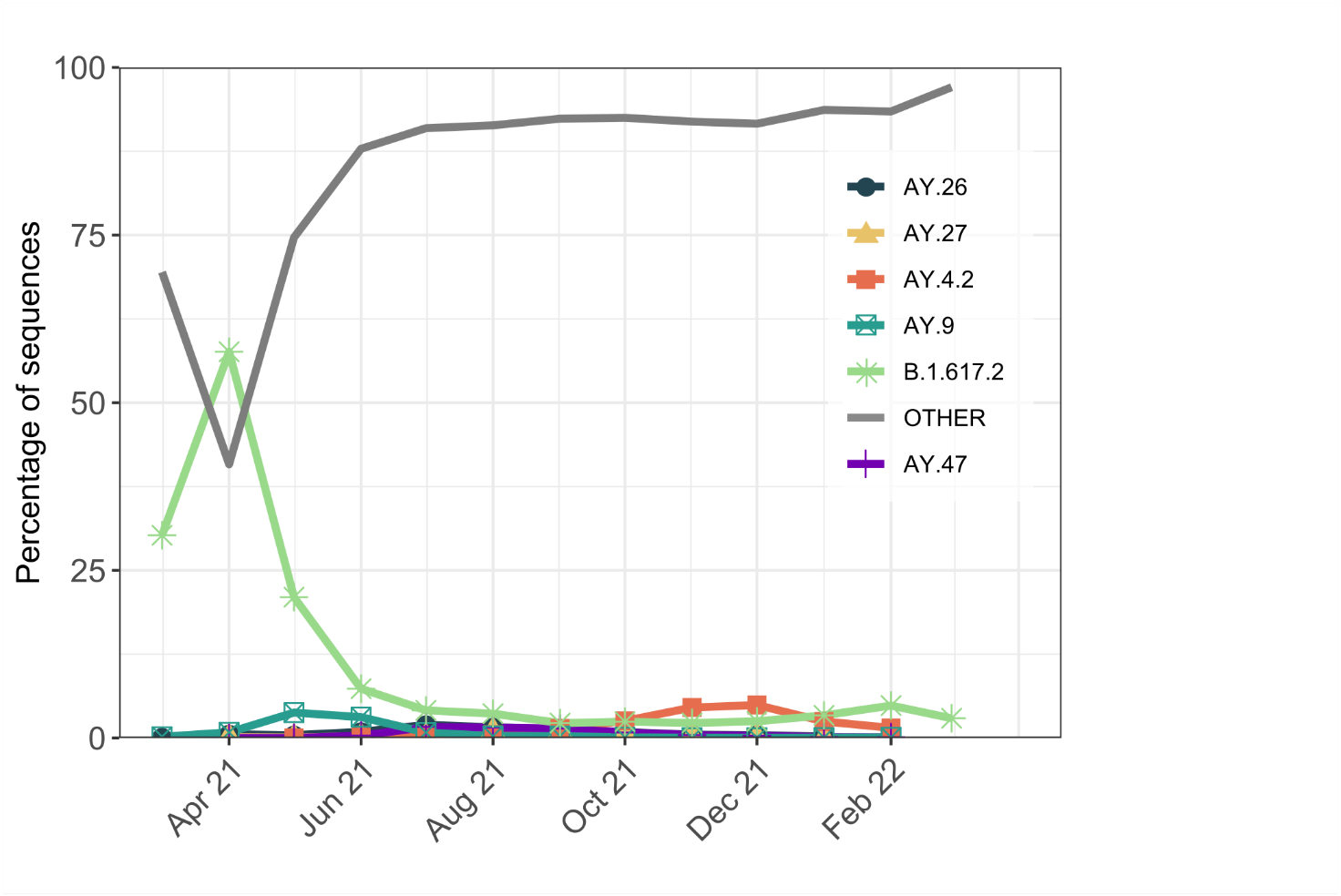


**Fig A. Temporal distribution of Delta sub-lineages.** Percentage of SARS-CoV-2 sequences designated as parental B.1.617.2 and its sub-lineages (designated as AY).


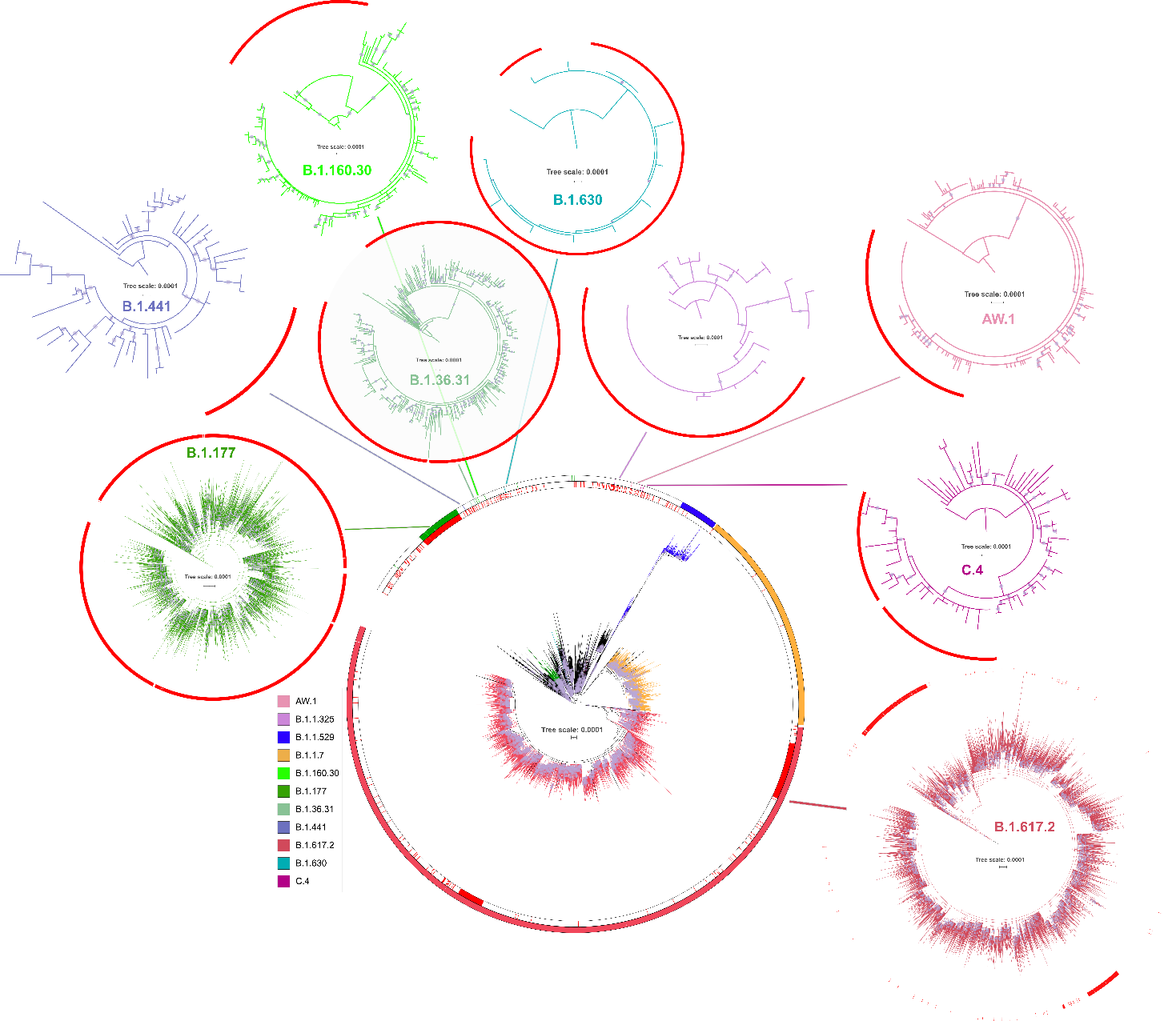


**Fig B.** **Recurrent emergence of S:A222V in different SARS-CoV-2 scenarios in the G clade.** The phylogeny in the middle is composed by 11,166 sequences belonging to G clade, in the red inner circle are annotated sequences with S:A222V, the external circle indicates the PANGO linages of interest coloured in the legend. From the phylogeny of G clade in the middle emerges another 9 phylogenies belonging to PANGO lineages with more than 10% sequences with S:A222V. The red circle indicates sequences with S:A222V. Each circle in branches correlates with the bootstrap value; only bootstraps from 70 to 100 are represented. Each scale bar indicates the number of nucleotide substitutions per site.


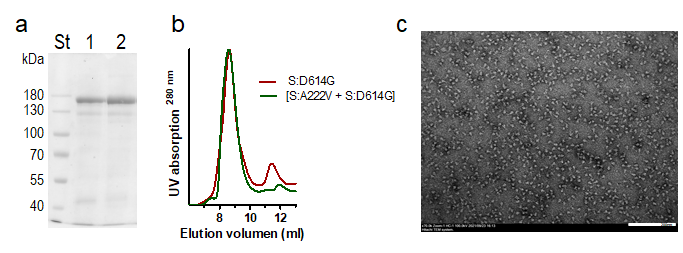


**Fig C. Protein purification and quality analysis. (a)** SDS-PAGE (Coomasie Staining) of the (1) S:D614G or (2) S:[A222V + S:D614G] spike variants. St, Pageruler Prestained Protein Ladder (Thermofisher Scientific) with masses (in kDa) indicated at the sides. (**b)** Size-exclusion chromatographic (SEC) profiles (UV absorption) of the indicated spike variants. (**c)** Electron micrograph of negatively stained particles from SEC fraction of D614G Spike protein, with scale bar = 200 nm.


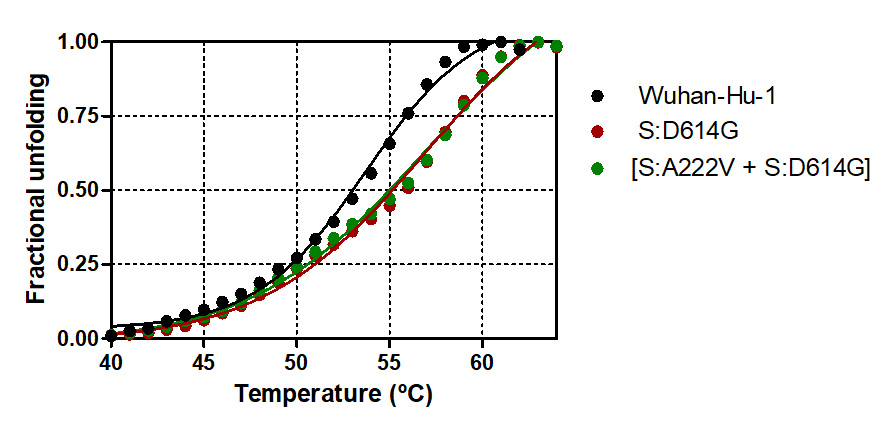


**Fig D.** **Thermofluor assays (means of three replicas) of the indicated SARS-CoV-2 S protein mutants.** Error bars are represented (too small to be seen).


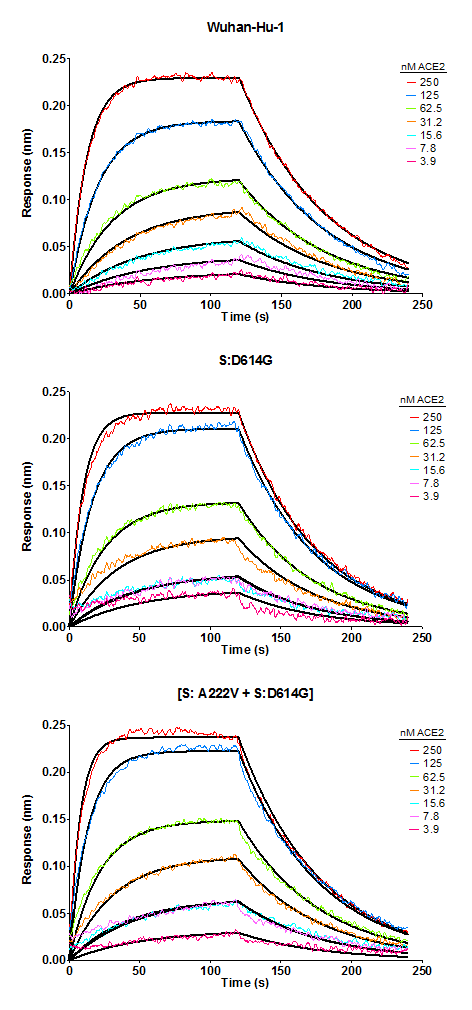


**Fig E. Kinetic analysis of spike interaction to ACE2 measured by biolayer interferometry (BLI).** Wuhan-Hu-1, S:D614G and [S:A222V + S:D614G]. The association of the different spike variants to ACE2 was carried out for 120s at various concentrations in a two-fold dilution series from 250 to 3.9 nM prior to dissociation for 120s. Curves were plotted using GraphPad Prism 6 for macOSX and fitting was performed using a 1:1 binding model in the data analysis HT software (Fortebio). Calculation of on-rates (K_on_), off-rates (K_off_) and affinity constants (K_D_) were computed using a global fit applied to all data. Raw data are coloured representations and fitting models are shown in black. Results are summarized in **Table 1** of the main text.


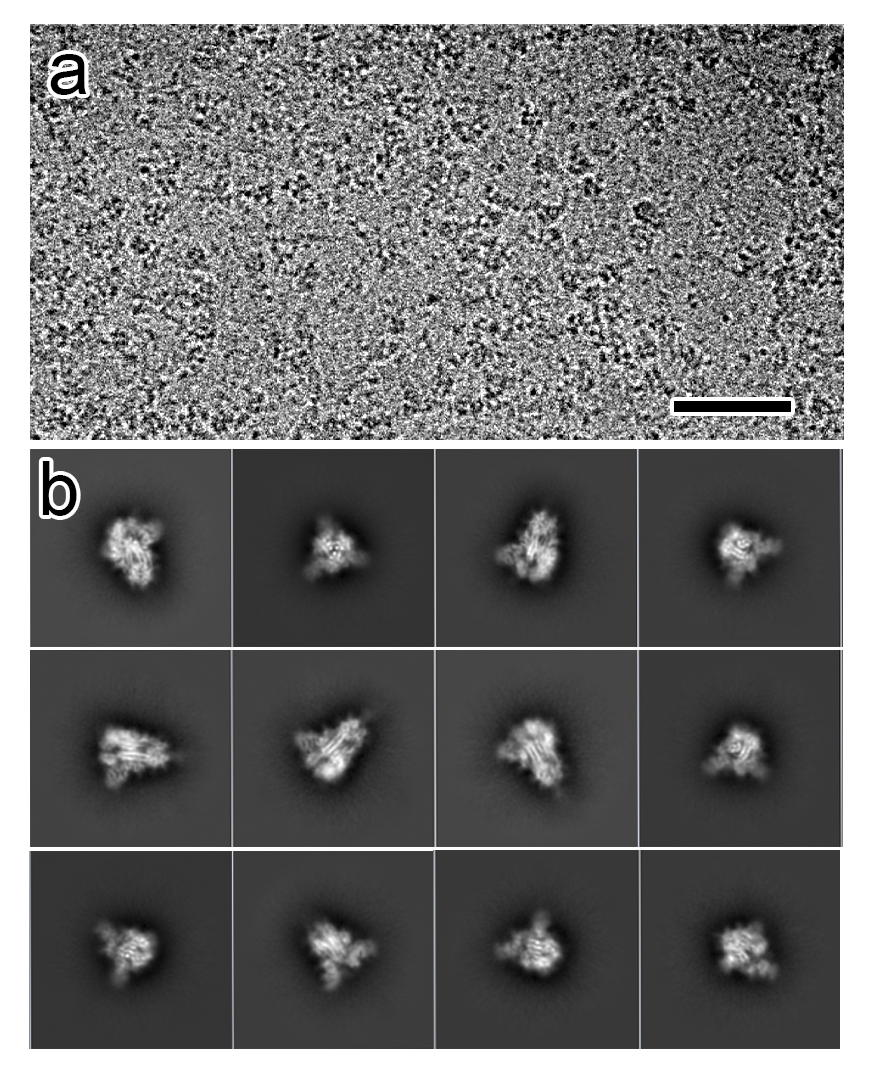


**Fig F. Cryo-EM data of [S:A222V + S:D614G]**. (**a**) Representative micrograph of the [S:A222V + S:D614G] mutant. Bar = 50 nm. (**b**) Set of representative side and top view class averages obtained after reference-free 2D classification of automatically picked and extracted particles.


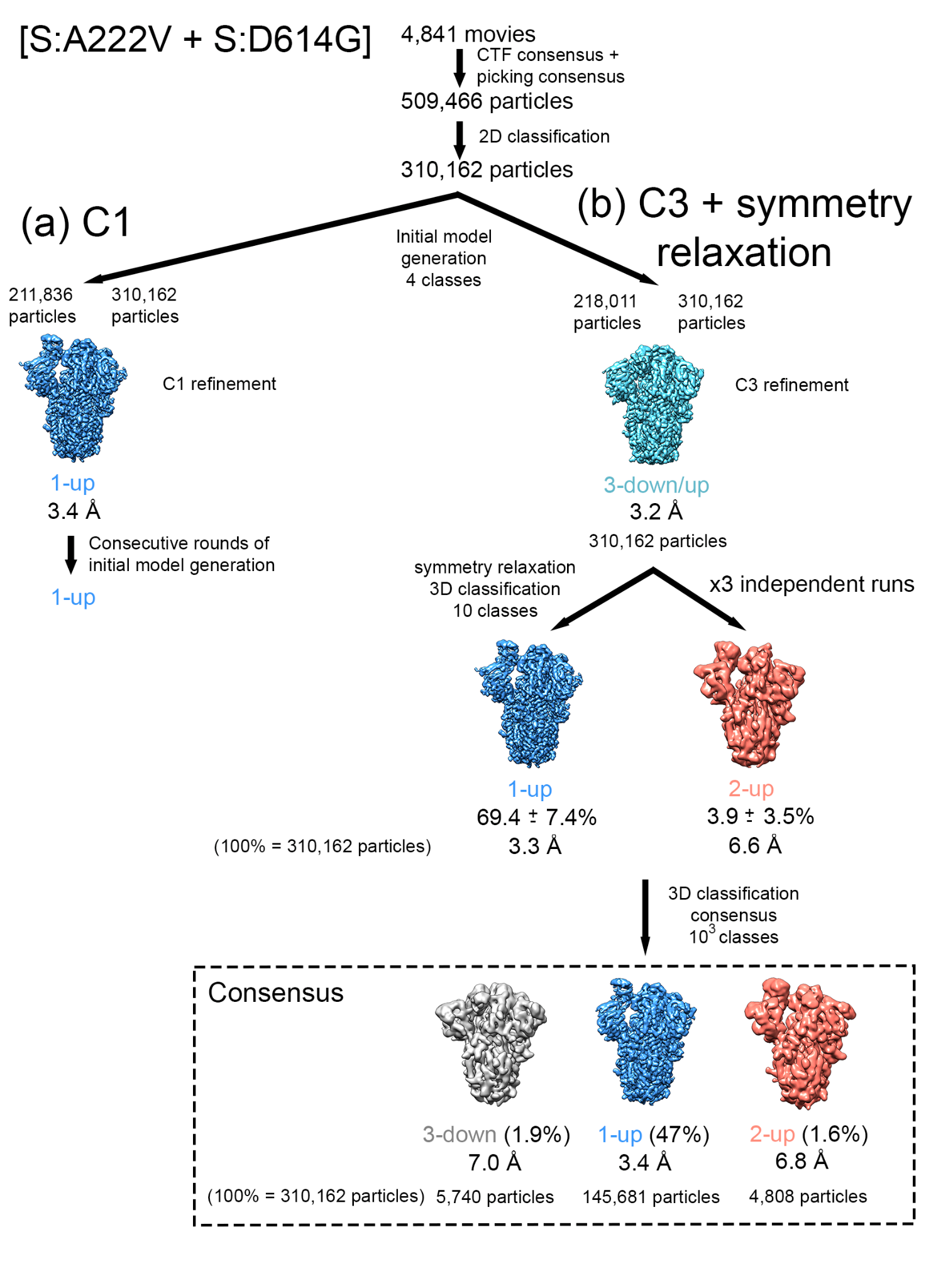


**Fig G. Cryo-EM image processing workflow for [S:A222V + S:D614G].** The initial dataset of 4,841 movies was subjected to motion-correction alignment, followed by CTF correction, automatic particle picking and subsequent extraction of 509,466 particles. After reference-free 2D classification, 310,162 particles were selected. Then, particles were subjected to initial model generation in 4 classes without (**a**) or with C3 symmetry imposition (**b**). In both image processing approaches, datasets considering unique or combined classes were refined to a resolution of 3.4 Å (**a**) and 3.2 Å (**b**). In (**a**), all of the maps obtained considering different classes and thus from different numbers of particles (211,836 or 310,162 particles) consisted of molecules with a 1-up conformation (blue). Consecutive rounds of initial model generation did not result in different RBD conformations, nor improvement of resolution. In (**b**), all of the maps obtained from different classes, each containing a different number of particles (218,011 or 310,162 particles), consisted of a 3-down/up mixed conformation (light blue) as a consequence of the symmetry imposition. The 310,162 particles dataset was then subjected to a first round of 3D classification with a symmetry relaxation implementation (**Methods**) in 10 classes and resulted in a main 1-up conformation (69%, blue) but also a minor 2-up conformation (7%, red). This 3D classification was repeated three times (mean and standard deviation are indicated) and subjected to the 3D classification consensus protocol. Note the power of the consensus approach (Sorzano et al. 2021), where a total of 10x10x10 = 1000 subclasses has been considered, selecting only those groups of particles that were always classified together (in other words, that they were stable clusters, coming together in three independent runs of classification). Naturally, results are considered to be stable only after consensus, presenting a % of occupancy much more reproducible than just after a first round. An initial model was generated for each selected class/conformation and subsequently refined to a resolution of 3.3-3.4 Å (1-up, blue), 6.6-6.8 Å (2-up, red) and 7 Å (3-down, grey). All of the maps shown were sharpened with DeepEMhancer (Sanchez-Garcia et al. 2021). Please note that the percentages shown do not add up to 100% (310,162 particles) in either analysis because some maps did not seem to be regular spikes and were assumed to contain junk particles.


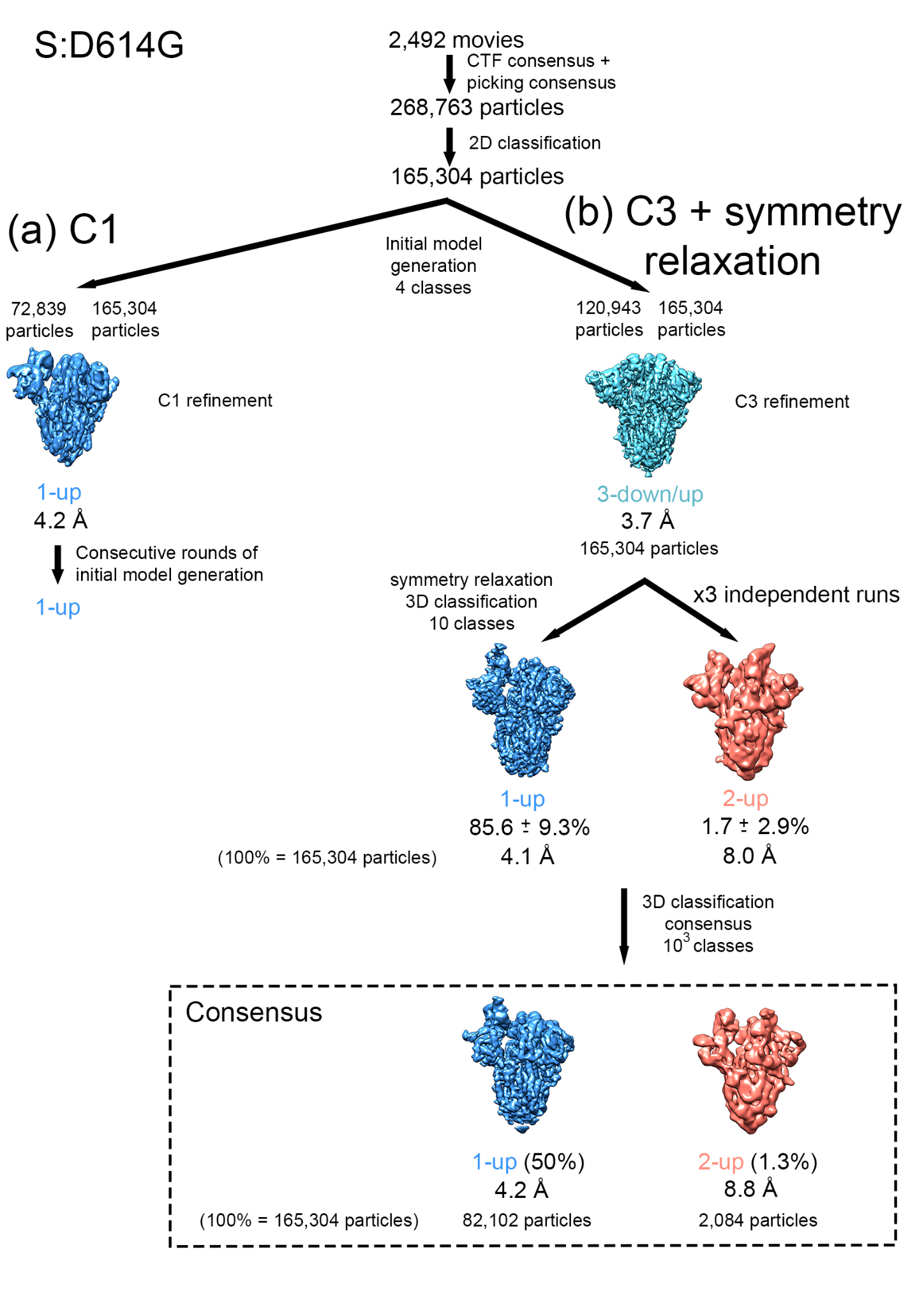


**Fig H.** **Cryo-EM image processing workflow for S:D614G.** The initial dataset of 2,492 movies was subjected to motion-correction alignment, followed by CTF correction, automatic particle picking and subsequent extraction of 268,763 particles. After reference-free 2D classification, 165,304 particles were selected. Then, particles were subjected to initial model generation in 4 classes without (**a**) or with C3 symmetry imposition (**b**). In both image processing approaches, datasets considering unique or combined classes were refined to a resolution of 4.2 Å (**a**) and 3.2 Å (**b**). In (**a**), all of the maps obtained considering different classes and thus from different numbers of particles (72,839 or 165,304 particles) consisted of molecules with a 1-up conformation (blue). Consecutive rounds of initial model generation did not result in different RBD conformations, nor improvement of resolution. In (**b**), all of the maps obtained from different classes and thus from different numbers of particles (120,943 or 165,304 particles), consisted of a 3-down/up mixed conformation (light blue) as a consequence of the symmetry imposition. The 165,304 particles dataset was then subjected to 3D classification with a symmetry relaxation implementation (**Methods**) in 10 classes and resulted in a main 1-up conformation (75%, blue) but also a minor 2-up conformation (5%). This 3D classification was repeated three times (mean and standard deviation are indicated) and subjected to the 3D classification consensus protocol as for the double mutant. An initial model was generated for each selected class/conformation and subsequently refined to a resolution of 4.1-4.2 Å (1-up, blue) and 8.0-8.8 Å (2-up, red). All of the maps shown were sharpened with DeepEMhancer (Sanchez-Garcia et al. 2021). Please note that the percentages shown do not add up to 100% (165,304 particles) in either analysis because some maps did not look like regular spikes and were assumed to contain junk particles.


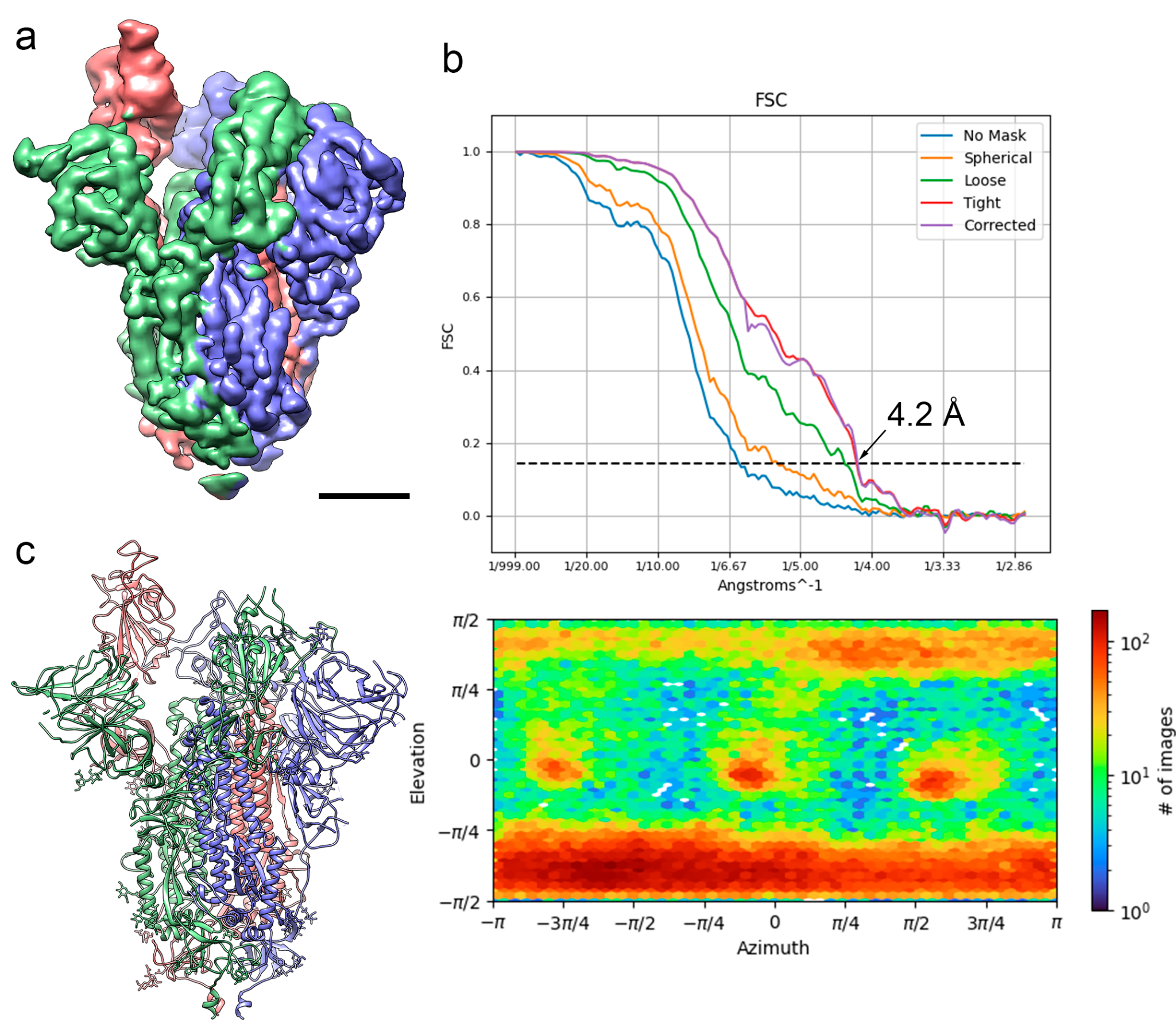


**Fig I. Cryo-EM of S:D614G. (a**) Side view of the cryo-EM density map of the S:D614G mutant obtained after consensus of three independent runs of symmetry relaxation, followed by initial model generation, refinement without symmetry imposition and deepEMhancer map sharpening, shown at 3 σ. Bar = 30 Å. (**b**) Fourier Shell Correlation (FSC) resolution curve (top) shown as the regular cryoSPARC global FSC resolution output, which includes no mask and different masks. Resolution based on the gold standard 0.143 criterion is 4.2 Å. Angular distribution coverage profile (bottom). (**c**) Atomic model of the D614G mutant shown as ribbon diagrams. S protein subunits are coloured in blue (A chain), red (B chain) and green (C chain). Glycan molecules are shown as stick diagrams and coloured according to their corresponding subunit.


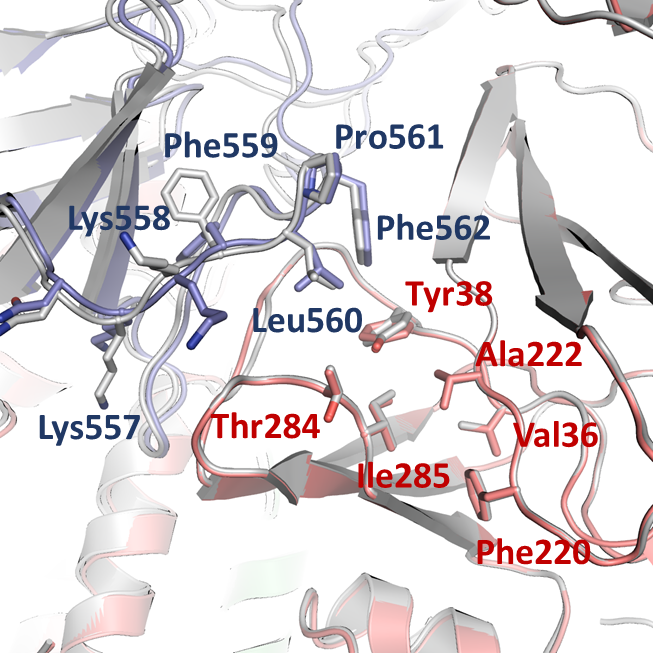


**Fig J. Comparison of the two structures at the region where S:A222V mutation is located.** The NTD_B_ and the CTD1_A_ of the double mutant [S:A222V + S:D614G] are coloured in salmon and blue respectively, while in the single mutant S:D614G, both subunits are coloured in grey. Side chains from residues surrounding the mutated residue are shown in sticks and indicated.


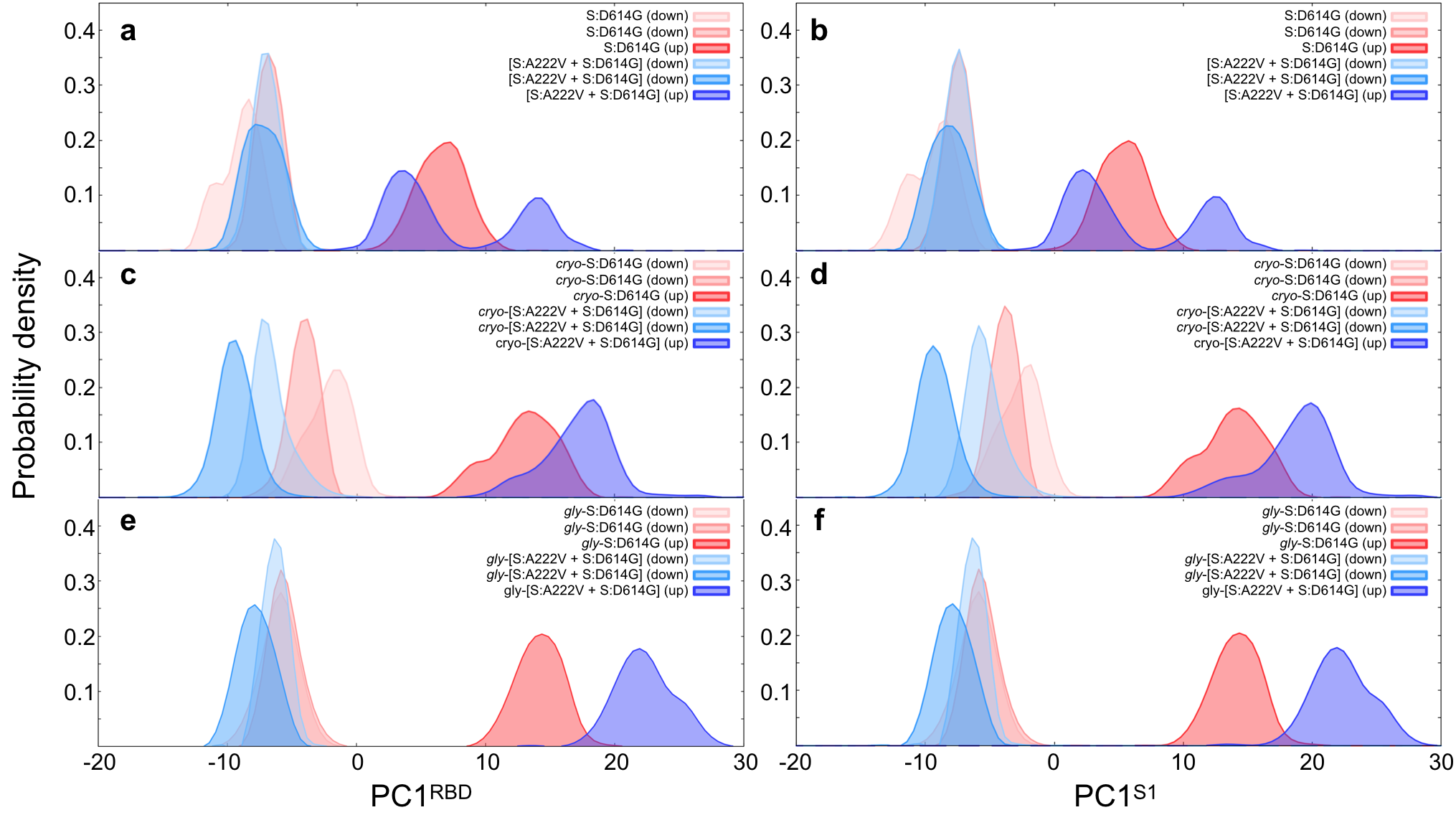


**Fig K. Continuous population densities for multiple subunits along the PC1.** Continuous population densities for multiple subunits along the first eigenvector for the PCA based on RBD (residues; 330-530; PC1^RBD^; **a, c, e**) and S1 (residues 30-650; PC1^S1^; **b, d, f**) of the MD-simulated 1-up (UDD) trimeric ensemble relative to the (**a**-**b**) glycan-free, (**c**-**d**) cryo-EM and (**e**-**f**) fully glycosylated S:D614G (as shades of red) and [S:A222V + S:D614G] (as shades of blue).


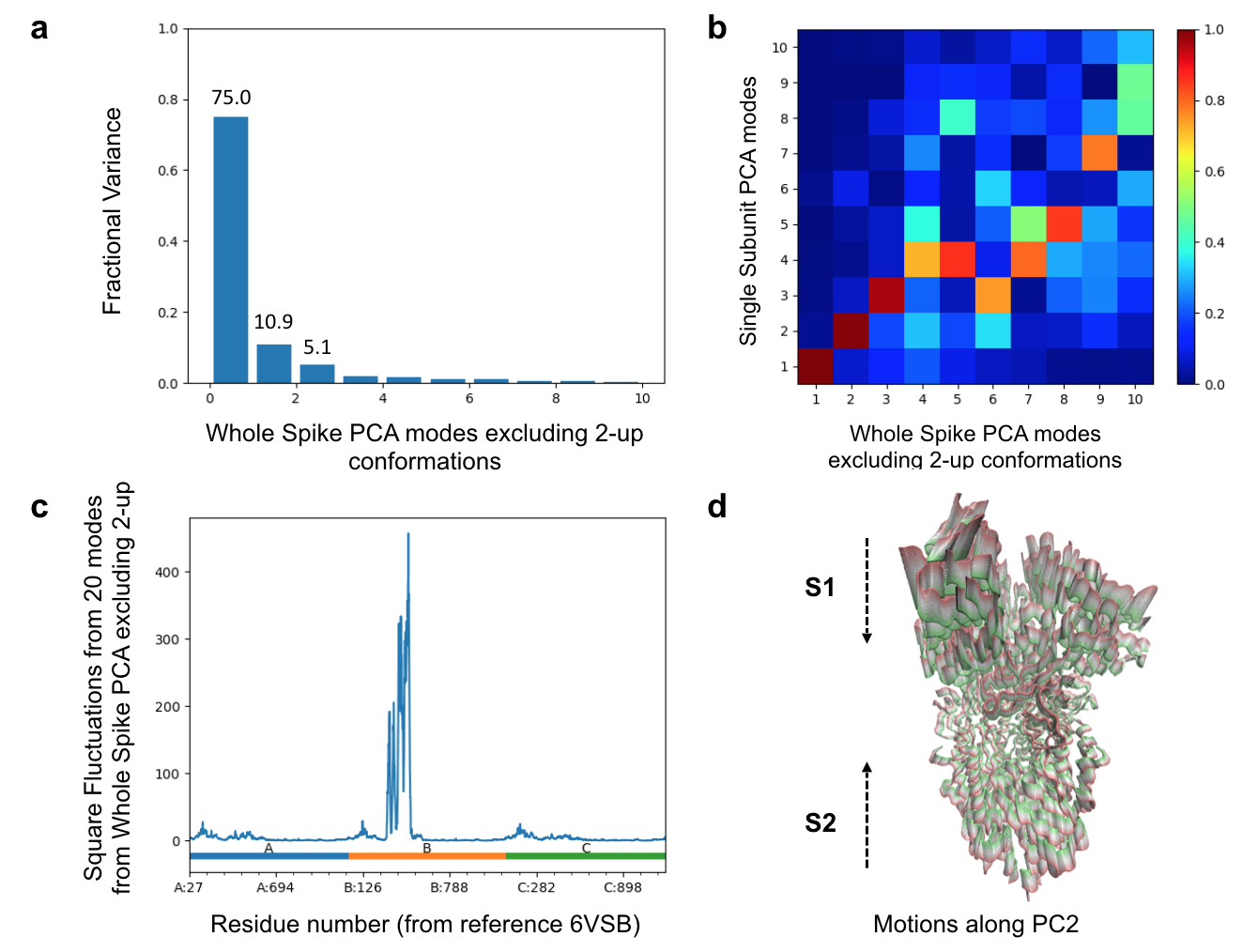


**Fig L. PCA of whole experimental S:D614G spike structures excluding 2-up conformations.** **(a)** Fractional variance contributed by each of the first 10 modes from the same whole spike PCA. Percentages (values multiplied by 100) are shown for the first three modes, which contribute more than 5 % (0.05) of the variation. **(b)** A matrix of correlation cosine overlaps comparing mode eigenvectors from a PCA performed using only the subunit whose RBD undergoes the transition to 1 up (ordinate) and that using the whole spike but excluding 2-up conformations (sliced to only include corresponding residues for the comparison; abscissa). The matrix is coloured from low overlaps in dark blue to high overlaps in dark red via cyan, green, yellow and orange as shown in the colour bar on the right. **(c)** Square fluctuations from the first 20 modes of variation from the same whole spike PCA weighted by the fractional variances. Residue numbers and chains are labelled as in our cryo-EM structures, allowing identification of motions of the dominant RBD (residues 330-530 of chain B) as well as other NTDs and RBDs. **(D)** Side view of the spike showing compression and stretching motions observed in whole spike PC2 using a range of states spanning 4 A from the average sone extreme in green (compressed state) to the other in red (stretched state).


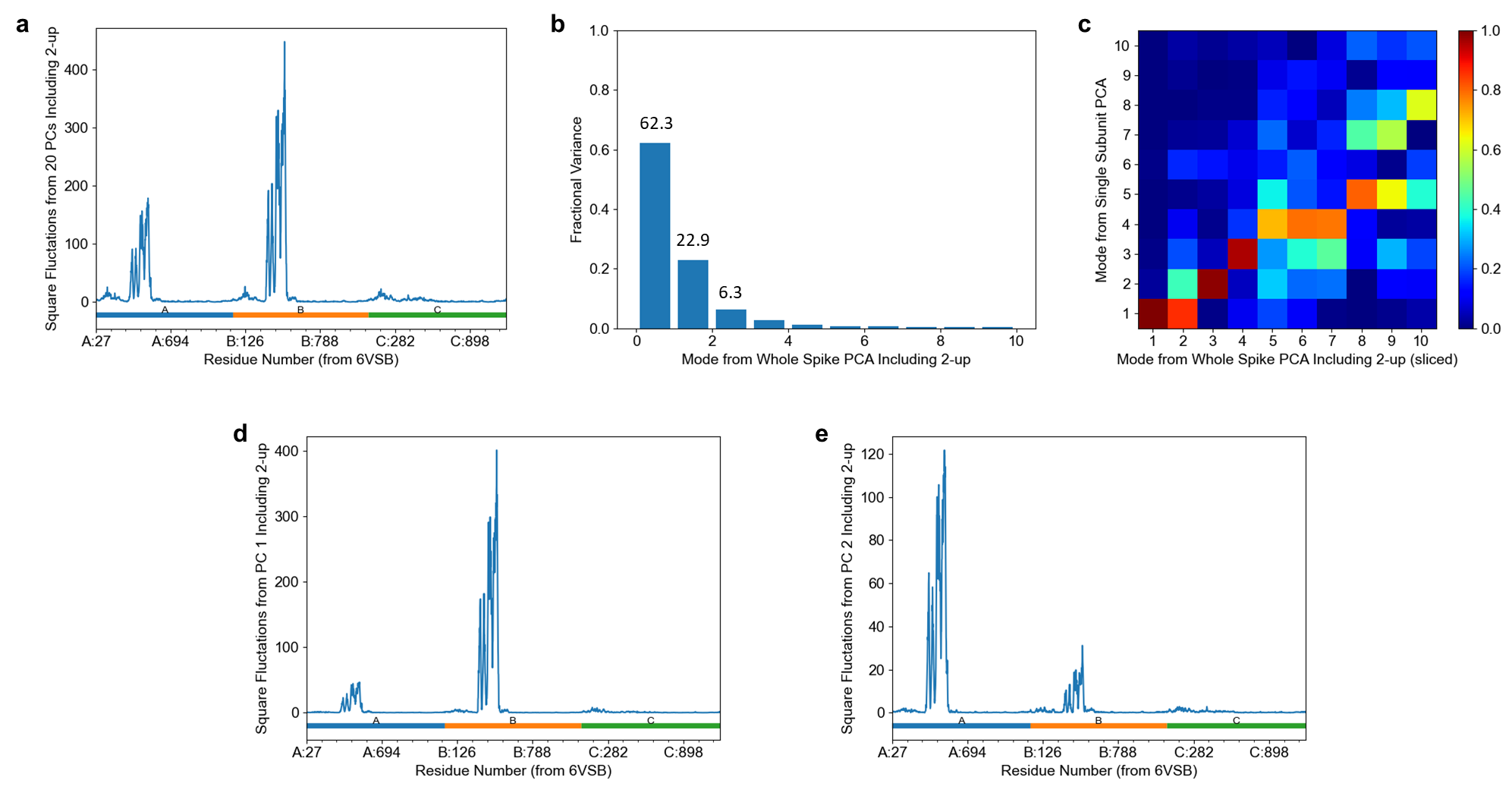


**Fig M.** **PCA results for whole S:D614G PDB structures including 2-up conformations.** **(a)** Square fluctuations from the first 20 modes of variation from the PCA from the whole spike excluding 2-up conformations weighted by the fractional variances. Residue numbers and chains are labelled as in our cryo-EM structures, allowing identification of motions of the dominant RBD (residues 330-530 of chain B) as well as other NTDs and RBDs. **(b)** Fractional variance contributed by each of the first 20 modes of the PCA from the whole spike excluding 2-up conformations. Percentages (values multiplied by 100) are shown for the first three modes, which contribute more than 5 % (0.05) of the variation. **(c)** A matrix of correlation cosine overlaps comparing mode eigenvectors from a PCA performed using only the subunit whose RBD undergoes the transition to 1 up (ordinate) and that using the whole spike including 2-up conformations in the covariance calculation (sliced to only include corresponding residues for the comparison; abscissa). The matrix is coloured from low overlaps in dark blue to high overlaps in dark red via cyan, green, yellow and orange as shown in the colour bar on the right. **(d-e)** Square fluctuations from PC1 **(d)** and PC2 **(e)**, showing dominance of the first RBD (chain B) and second RBD (chain A), respectively.


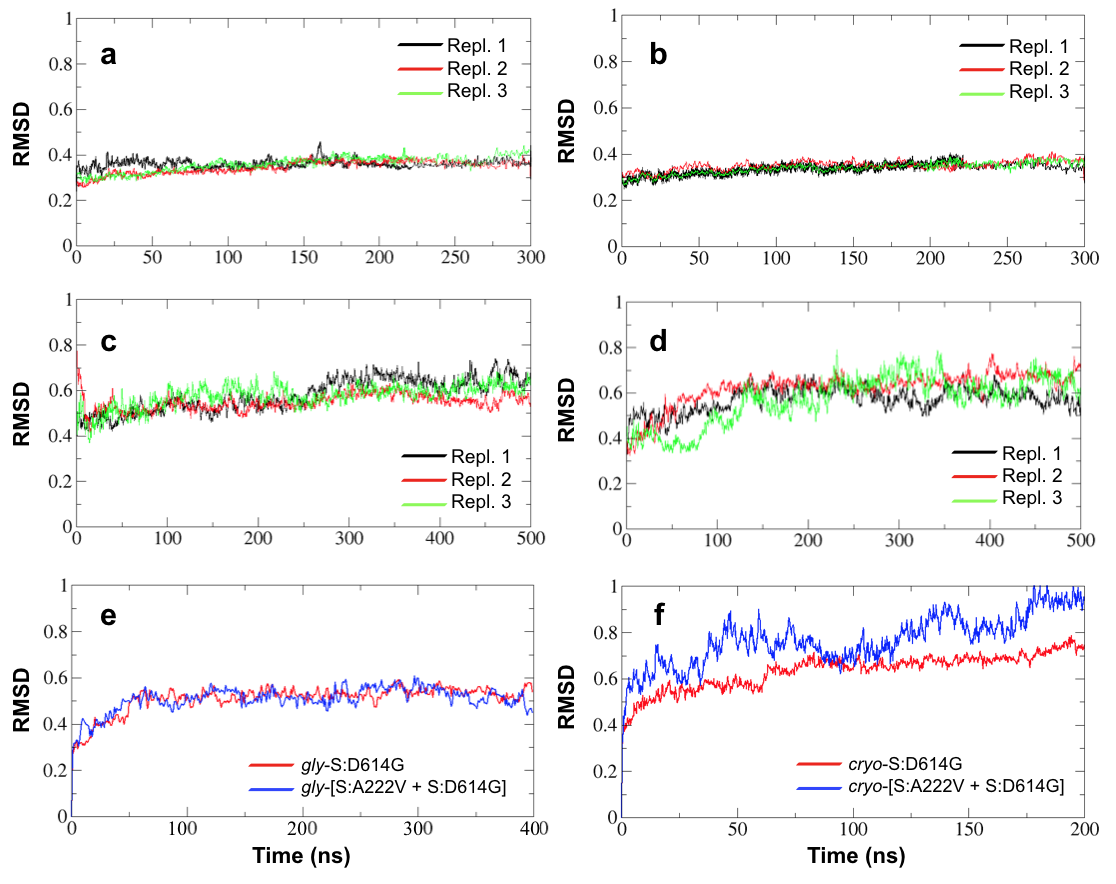


**Fig N. RMSD analysis.** Root-mean-square deviation (RMSD, nm) analysis of the MD trajectories (in triplicate) for the S:D614G (**a**, **c**) and [S:A222V + S:D614G] (**b**, **d**) mutants of the SARS-CoV-2 Spike mutants in the closed, 3-down (DDD; **a**, **b**) and open, 1-up (UDD; **c**, **d**) states respectively based on 6VXX and 6VSB. The same analysis for (**e**) the fully glycosylated mutants based on 6VSB and for (**f**) the (glycan-free) cryo-EM mutants in the open, 1-up state is also reported.

**Tables**

**Table A.** Frequencies of sequences with A222V for the different PANGO lineages. Only first 10 rows shown with highest A222V sequence counts; full table in a separate .xlsx file (**S1 Table**).

| **VOC** | **Clade** | **PANGO lineage** | **Nº of Sequences with A222V in PANGO lineage** | **Nº of Sequences in PANGO lineages** | **Percentage of A222V in PANGO lineages** |
| --- | --- | --- | --- | --- | --- |
| B.1.617.2 | GK | AY.4.2 | 67224 | 67296 | 99.89 |
| B.1.177 | GV | B.1.177 | 57875 | 58393 | 99.11 |
| B.1.617.2 | GK | AY.9.2 | 34663 | 41703 | 83.12 |
| B.1.617.2 | GK | AY.26 | 31623 | 32097 | 98.52 |
| B.1.617.2 | GK | AY.47 | 29203 | 29609 | 98.63 |
| B.1.617.2 | GK | AY.4.2.2 | 21905 | 21974 | 99.69 |
| B.1.617.2 | GK | AY.27 | 17610 | 17624 | 99.92 |
| B.1.617.2 | GK | AY.75 | 16790 | 17097 | 98.20 |
| B.1.617.2 | GK | AY.4.2.1 | 13267 | 14060 | 94.36 |

**Table B.** Cryo-EM data collection, refinement and validation statistics. Upper part: General parameters used during cryo-EM data collection and image processing. Lower part: general statistics obtained from refinement and validation of the atomic model and cryo-EM density map.

|  | **[S:A222V + S:D614G]** | | | **[S:D614G]** | |
| --- | --- | --- | --- | --- | --- |
|  | **3-down**  **EMD-13918** | **1-up**  **EMD-13916**  **PDB: 7QDG** | **2-up**  **EMD-13917** | **1-up**  **EMD-13919**  **PDB: 7QDH** | **2-up**  **EMD-13920** |
| **Data collection and map reconstruction** |  |  |  |  |  |
| Microscope | FEI Talos Artica | FEI Talos Artica | FEI Talos Artica | FEI Talos Artica | FEI Talos Artica |
| Detector | Falcon 3 | Falcon 3 | Falcon 3 | Falcon 3 | Falcon 3 |
| Magnification | 120,000x | 120,000x | 120,000x | 120,000x | 120,000x |
| Voltage (kV) | 200 | 200 | 200 | 200 | 200 |
| Electron exposure (e^-^/Å^2^) | 32.4 | 32.4 | 32.4 | 32.4 | 32.4 |
| Exposure per frame (e^-^/Å^2^) | 0.54 | 0.54 | 0.54 | 0.54 | 0.54 |
| Defocus range (µm) | -0.3 to -3.5 | -0.3 to -3.5 | -0.3 to -3.5 | -0.3 to -3.5 | -0.3 to -3.5 |
| Pixel size (Å) | 0.85 | 0.85 | 0.85 | 0.85 | 0.85 |
| Micrographs collected (no.) | 4,841 | 4,841 | 4,841 | 2,492 | 2,492 |
| Initial particles (no.) | 509,466 | 509,466 | 509,466 | 268,763 | 268,763 |
| Final particles (no.) | 5,740 | 145,681 | 4,808 | 82,102 | 2,084 |
| Symmetry imposed | C3 + symmetry  relaxation (C1) | C3 + symmetry  relaxation (C1) | C3 + symmetry  relaxation (C1) | C3 + symmetry  relaxation (C1) | C3 + symmetry  relaxation (C1) |
| Map resolution (Å)  (gold standard FSC=0.143) | 7.0 | 3.4 | 6.8 | 4.2 | 8.0 |
| Map resolution range (Å) | 6.7 – 15.0 | 3.2 – 8.0 | 6.4 – 14.0 | 3.9 – 9.0 | 7.5 – 13.0 |
| **Model building, refinement and validation** |  |  |  |  |  |
| Model resolution (Å) |  | 3.4 |  | 4.2 |  |
| Average model to map correlation coefficient |  | 0.71 |  | 0.42 |  |
| Map sharpening B factor (Å^2^) |  | deepEMhancer |  | deepEMhancer |  |
| Model composition |  |  |  |  |  |
| Non-hydrogen atoms |  | 25,557 |  | 25,703 |  |
| Protein residues |  | 3,193 |  | 3,219 |  |
| Ligands |  |  |  |  |  |
| NAG |  | 47 |  | 44 |  |
| *B* factors |  | 139.52 |  | 204.35 |  |
| Protein |  | 138.51 |  | 202.63 |  |
| Ligands |  | 177.88 |  | 274.59 |  |
| R.m.s. deviations (Å^2^) |  |  |  |  |  |
| Bond lengths (Å) |  | 0.01 |  | 0.01 |  |
| Bond angles (˚) |  | 1.53 |  | 1.56 |  |
| Validation |  |  |  |  |  |
| MolProbity score |  | 3.20 |  | 3.25 |  |
| Clashscore |  | 53.77 |  | 61.99 |  |
| Rotamer outliers (%) |  | 7.20 |  | 7.77 |  |
| C-beta outliers (%) |  | 0.47 |  | 0.53 |  |
| Rama Z-score |  | 0.28±0.14 |  | 0.10±0.14 |  |
| Ramachandran plot |  |  |  |  |  |
| Favored (%) |  | 95.03 |  | 95.61 |  |
| Allowed (%) |  | 4.59 |  | 4.01 |  |
| Outliers (%) |  | 0.38 |  | 0.38 |  |

**Table C.** Interaction between RBDs and NTDs domains from different subunits.

|  | **Interaction Surface (Å^2^)** | |
| --- | --- | --- |
|  | **[S:A222V + S:D614G]** | **S:D614G** |
| **Subunits A and B** | | |
| TOTAL | 1,011.3 | 882.4 |
| RBD_A_-NTD_B_ | 793.8 | 732.7 |
| RBD_A_-RBD_B_ | 217.5 | 149.7 |
| **Subunits B and C** | | |
| TOTAL (RBD_B_-NTD_C_) | 746.7 | 748.2 |
| **Subunits A and C** | | |
| TOTAL | 818.2 | 771.8 |
| NTD_A_-RBD_C_ | 744.2 | 717.7 |
| RBD_A_-RBD_C_ | 74 | 54.1 |

**Table D.** Detailed description of the 3-down (DDD) and 1-up (UDD) models of the SARS-CoV-2 spike mutants simulated in this study.

| **Glycan-free mutants from 6VXX/6VSB** | | | | |
| --- | --- | --- | --- | --- |
| **MODEL** | **N° atoms for the solvated systems** | **N° of TIP3P water molecules** | **N° of Na^+^ ions to neutralize** | **Simulated time**  **(ns)** |
| DDD_S:D614G_ |  |  |  |  |
| Replica 1 | 553,842 | 167,613 | 3 | 300 |
| Replica 2 | 553,842 | 167,613 | 3 | 300 |
| Replica 3 | 553,842 | 167,613 | 3 | 300 |
| DDD_[S:A222V + S:D614G]_ |  |  |  |  |
| Replica 1 | 553,860 | 167,613 | 3 | 300 |
| Replica 2 | 553,860 | 167,613 | 3 | 300 |
| Replica 3 | 553,860 | 167,613 | 3 | 300 |
| UDD_S:D614G_ |  |  |  |  |
| Replica 1 | 735,249 | 228,082 | 3 | 500 |
| Replica 2 | 735,249 | 228,082 | 3 | 500 |
| Replica 3 | 735,249 | 228,082 | 3 | 500 |
| UDD_[S:A222V + S:D614G]_ |  |  |  |  |
| Replica 1 | 735,258 | 228,079 | 3 | 500 |
| Replica 2 | 735,258 | 228,079 | 3 | 500 |
| Replica 3 | 735,258 | 228,079 | 3 | 500 |
| **Glycosylated mutants from 6VSB** | | | | |
| **SYSTEM** | **N° atoms for the solvated systems** | **N° of TIP3P water molecules** | **N° of K^+^/Cl^-^ ions to neutralize** | **Simulated time**  **(ns)** |
| *gly-*UDD_S:D614G_ |  |  |  |  |
| Replica 1 | 746,558 | 226,799 | 709/694 | 400 |
| *gly-*UDD_[S:A222V + S:D614G]_ |  |  |  |  |
| Replica 1 | 746,558 | 226,799 | 709/694 | 400 |
| **cryo-EM mutants of this work** | | | | |
| **SYSTEM** | **N° atoms for the solvated systems** | **N° of TIP3P water molecules** | **N° of K^+^/Cl^-^ ions to neutralize** | **Simulated time**  **(ns)** |
| *cryo-*UDD_S:D614G_ |  |  |  |  |
| Replica 1 | 826,453 | 257,517 | 767/767 | 200 |
| *cryo-*UDD_[S:A222V + S:D614G]_ |  |  |  |  |
| Replica 1 | 826,510 | 257,530 | 767/767 | 200 |

**Table E. Mutational free energy analysis.** Alchemical free energy calculations for the S:D614G and [S:A222V + S:D614G] mutants in the open, 1-up conformation based on 6VSB and our cryo-EM data. The obtained relative free energy values for the analysed systems indicate that the impact of the S:A222V mutation on the free energy of RBD opening is too small to establish a significant preference for either of the conformational states of the RBD.

| **Conformation** | ***gly*-UDD, S:A222V^a^** | ***cryo*-UDD, S:A222V^a^** | ***gly*-UDD, S:D614G^b^** |
| --- | --- | --- | --- |
| **Open** | 0.58 | 0.15 | 0.00 |
| **Closed (1)** | 0.45 | 0.00 | 4.15 |
| **Closed (2)** | 0.00 | 0.17 | 2.26 |

^a^ Relative values for S:A222V calculated on the background of S:D614G.

^b^ Relative values for S:D614G calculated on the background of Wuhan-Hu-1.

All values (in kcal/mol) are reported in relative terms (subtracting the lowest value).

**Table F.** Experimental structures of the SARS-CoV-2, S:D614G spike used in PCA analysis.

|  | **PDB ID** | **State^a^** | **CS Mutations** | **Proline Mutations** | **Chain Order** | **Reference** |
| --- | --- | --- | --- | --- | --- | --- |
| **1** | **6ZWV** | 3-down | - | - | BAC | Ke et al. 2020 |
| **2** | **7BNM** | 3-down | RSAS | PP | BCA | Benton et al. 2021 |
| **3** | **7BNN** | 1-up | RSAS | PP | BCA | Benton et al. 2021 |
| **4** | **7BNO** | 2-up | RSAS | PP | BCA | Benton et al. 2021 |
| **5** | **7KDI** | 3-down | - | - | BCA | Gobeil et al. 2021 |
| **6** | **7KDJ** | 1-up | - | - | BCA | Gobeil et al. 2021 |
| **7** | **7KE4** | 3-down | GSAS | - | BCA | Gobeil et al. 2021 |
| **8** | **7KE6** | 3-down | GSAS | - | BCA | Gobeil et al. 2021 |
| **9** | **7KE7** | 3-down | GSAS | - | BCA | Gobeil et al. 2021 |
| **10** | **7KE8** | 3-down | GSAS | - | BCA | Gobeil et al. 2021 |
| **11** | **7KE9** | 1-up | GSAS | - | BCA | Gobeil et al. 2021 |
| **12** | **7KEA** | 1-up | GSAS | - | BCA | Gobeil et al. 2021 |
| **13** | **7KEB** | 1-up | GSAS | - | BCA | Gobeil et al. 2021 |
| **14** | **7KEC** | 1-up, 1-I | GSAS | - | BCA | Gobeil et al. 2021 |
| **15** | **7KRQ** | 3-down | - | - | ABC | Zhang et al. 2021 |
| **16** | **7KRR** | 1-up | - | - | ABC | Zhang et al. 2021 |
| **17** | **7KRS** | 1-I | - | - | ABC | Zhang et al. 2021 |
| **18** | **7EAZ** | 1-up | GSAG | PP | ABC | Yang et al. 2021 |
| **19** | **7EB0** | 1-up | GSAG | PP | ABC | Yang et al. 2021 |
| **20** | **7EB3** | 1-up | GSAG | PP | ABC | Yang et al. 2021 |
| **21** | **7EB4** | 2-up | GSAG | PP | BCA | Yang et al. 2021 |
| **22** | **7EB5** | 2-up | GSAG | PP | ABC | Yang et al. 2021 |
| **23** | **7DX1** | 1-up | GSAS | PP | ABC | Yan et al. 2021 |
| **24** | **7DX2** | 1-up | -/digested | PP | ABC | Yan et al. 2021 |

^a^ “I” stands for Intermediate.

**Table G.** **Oligonucleotides used in this study.**

| Name | Sequence (5’-3’) |
| --- | --- |
| FW_ D614G_SPIKE | TCTTTACCAGGGCGTTAATTGTAC |
| RV_ D614G_SPIKE | ACAGCCACCTGGTTTGAC |
| FW_ A222V_SPIKE | GGGTTTTTCCGTACTAGAACCATTG |
| RV_ A222V_SPIKE | TGAGGTAAGTCGCGTAC |

**References**

1. Benton, D. J., A. G. Wrobel, C. Roustan, A. Borg, P. Xu, S. R. Martin, et al. 2021. “The effect of the D614G substitution on the structure of the spike glycoprotein of SARS-CoV-2.” *Proc Natl Acad Sci U S A.* 118 (9): e2022586118. DOI: 10.1073/pnas.2022586118.

2. Gobeil, S. M., K. Janowska, S. McDowell, K. Mansouri, R. Parks, K. Manne, et al. 2021. “D614G Mutation Alters SARS-CoV-2 Spike Conformation and Enhances Protease Cleavage at the S1/S2 Junction.” *Cell Rep.* 34 (2): 108630. DOI: 10.1016/j.celrep.2020.108630.

3. Ke, Z., J. Otón, K. Qu, M. Cortese, V. Zila, L. McKeane, et al. 2020. “Structures and distributions of SARS-CoV-2 spike proteins on intact virions.” *Nature.* 588 (7838): 498-502. DOI: 10.1038/s41586-020-2665-2.

4. Sanchez-Garcia, R., J. Gomez-Blanco, A. Cuervo, J. M. Carazo, C. O. S. Sorzano, and J. Vargas. 2021. “DeepEMhancer: a deep learning solution for cryo-EM volume post-processing.” *Commun Biol.* 4 (1): 874. DOI: 10.1038/s42003-021-02399-1.

5. Sorzano, C. O. S., A. Jiménez-Moreno, D. Maluenda, E. Ramírez-Aportela, M. Martínez, C. Cuervo, et al. 2021. “Image Processing in Cryo-Electron Microscopy of Single Particles: The Power of Combining Methods.” *Methods Mol Biol.* 2305:257-289. DOI: 10.1007/978-1-0716-1406-8_13.

6. Yan, R., Y. Zhang, Y. Li, F. Ye, Y. Guo, L. Xia, et al. 2021. “Structural basis for the different states of the spike protein of SARS-CoV-2 in complex with ACE2.” *Cell Res.* 31 (6). DOI: 10.1038/s41422-021-00490-0.

7. Yang, T. J., P. Y. Yu, Y. C. Chang, and S. D. Hsu. 2021. “D614G mutation in the SARS-CoV-2 spike protein enhances viral fitness by desensitizing it to temperature-dependent denaturation.” *J Biol Chem.* 29 (4): 101238. DOI: 10.1016/j.jbc.2021.101238.

8. Yurkovetskiy, L., X. Wang, K. E. Pascal, C. Tomkins-Tinch, T. P. Nyalile, Y. Wang, et al. 2020. “Structural and Functional Analysis of the D614G SARS-CoV-2 Spike Protein Variant.” *Cell.* 183 (3): 739-751.e8. DOI: 10.1016/j.cell.2020.09.032.

9. Zhang, J., Y. Cai, T. Xiao, J. Lu, H. Peng, S. M. Sterling, R, et al. 2021. “Structural impact on SARS-CoV-2 spike protein by D614G substitution.” *Science.* 372 (6541): 525-530. DOI: 10.1126/science.abf2303.
