# Supplementary material for: The structural role of SARS-CoV-2 genetic background in the emergence and success of spike mutations: The case of the spike A222V mutation
Source: PLoS Pathog. 2022 Jul 11;18(7):e1010631. doi: 10.1371/journal.ppat.1010631 (PMC9302720; doi:10.1371/journal.ppat.1010631)
Supplement: Attachment — Submitted filename: response_to_reviewers.pdf [file ppat.1010631.s004.pdf]

Dear Profs Heise and Haldar,

Thank you once more for your work on the review of this manuscript. We are including in this correspondence our responses to the referees' questions. We consider that we have addressed all the issues in a quite positive way.

In the next paragraphs, we will go through the different sections of the review report specifying what we have done in each of them (in italic and bold).

We are also including two text files, one showing where the changes have been introduced, and the other file containing clean text.

Additionally, we have introduced two changes worth mentioning at the onset. They are: (1) We propose a slight change of order of authors, so that Paula Ruiz-Rodriguez becomes a joint first author, considering the worth of new information that is now in the manuscript; (2) Unfortunately, we missed one author in the original submission, so that we are now including Rocio Arranz as co-author, from the cryo-EM facility. I can state that all authors in the original submission have been consulted and that they all have agreed on these changes, but if you need something else to proceed with them, please let me know.

We trust the manuscript will be now suitable for publication.

Yours sincerely

Prof. J.M. Carazo (on behalf of all authors)

Part I - Summary
Reviewer #1: This study described the S:A222V point mutation and its impact of such mutation. The authors performed serological, functional, structural and computational studies combined to reveal the circulation of such mutation and suggesting it doesn't affect vaccine effectiveness.

Reviewer #2: This manuscript from Ginex and colleagues characterizes the effect of the A222V substitution on the SARS-CoV-2 spike protein. This substitution has been selected multiple times in the evolution of SARS-CoV-2, suggesting that it provides an advantage to the virus. The investigators show that A222V does not provide escape from antibody-mediated neutralization, suggesting that it does not affect vaccine efficacy. Cryo-EM structures of an A222V/D614G spike protein and a D614G control spike protein reveal that the two structures are similar, as expected for a single amino acid substitution. MD simulations reveal that the A222V spike has a higher propensity to sample the open RBD state, which would improve attachment of the virus to the host cells.

The biophysical studies are performed to a high level, and the analyses are thorough. The cryo-EM studies are described well but I didn’t see a table for the cryo-EM model validation statistics, which needs to be included. As I am not an expert on MD simulations, I cannot comment on this aspect of the manuscript. Given that the MD results are key to supporting the major claim of the manuscript (that A222V increases RBD opening), it is difficult for this reviewer to determine whether the conclusions are supported by the data. The conclusion appears plausible, and would provide an advantage to the virus, supporting the repeated selection of A222V.

NOTE: A Table on validation statistics has been included (see Supplementary Table 2. Cryo-EM data collection, refinement and validation statistics). The topic is further addressed in Part III: Minor issues

---

Part II – Major Issues: Key Experiments Required for Acceptance

Reviewer #1: 1. The authors should define such mutation in the scope of lineage/sub-lineage/sub-clade. More importantly, a clear and detailed phylogenetic analysis (not that in Fig 1c) including these mutants within G clade should be provided.
2. The fitness related analysis for such mutants should be provided at least to show the natural selection of them. If the sequences are too many to be analyzed, I suggest to add some discussion for it.

Reviewer #2: (No Response)

We have followed the indications and we have addressed all the points raised

Section "Population dynamics of S:A222V through space and time" has been updated and expanded considerably. First, we have updated our analysis including the new sequences available between the previous submission (until September 2021) and the present submission (until April 2022). This new data is indicated as changes in the numbers and percentages in text, and the inclusion of data from the Omicron variant. Accordingly, we have modified Figure 1, and Supplementary Figure 1.

Second, we have expanded the analysis according to Reviewer 1 suggestions:

- ***i)*** ***We have defined the mutation S:A222V in the scope of lineage/sub-lineage/subclade by including a new table (Supplementary Table 1) that includes the number of sequences and percentage of sequences with S:A222V for each clade, sub-lineage and Variant of Concern.***
- ***ii)*** ***We have added a new phylogeny representing sequences with S:A222V within the G clade as suggested by the reviewer (Figure 1 panel a). Because clade G contains 4,993,996 sequences currently, we have reduced the dataset to 11,166 sequences, as described for the other dataset analysed, and kept the same temporal distribution by month. This new figure led to a reorganization of the old Figure 1, which now contains the new figure in panel a, and the old panel a as panel b. Therefore, panels b and c from Figure 1 are now Figure 2. Additionally, we have included a new supplementary figure (Supplementary Figure 2) which shows the expansion of sub-lineages of the Clade G phylogeny to further describe the results.***
- ***iii)*** ***As requested by reviewer 1, we have performed a selection analysis to address the selective advantage of the mutation. We show that S:A222V shows signatures of positive selection using different datasets. These results are included in a new paragraph at the end of the section and a new supplementary table (Supplementary Table 2) and a new panel included as Figure 1c, which contains the dN/dS values for each period. Additionally, we have included a reference that has already shown that S:A222V has been likely under adaptive selection (https://doi.org/10.1371/journal.pbio.3001115).***

Methods section "Sequence analysis" has been expanded and updated to "Sequence analysis and selection analysis".

Part III – Minor Issues: Editorial and Data Presentation Modifications
Please use this section for editorial suggestions as well as relatively minor

modifications of existing data that would enhance clarity.

Reviewer #1: (No Response)

Reviewer #2: A table containing the cryo-EM model validation statistics needs to be provided. The quality of the models, which were deposited in the Protein Data Bank with codes 7QDG and 7QDH, cannot be assessed without the table or PDB validation reports.

We have followed the indication and a new Table (Supplementary Table S2) on model validation statistics has been included in the manuscript.
